# Supplementary material for: Are carbon emissions trading and green financial instruments synergistic? -Comprehensive quantitative research based on content analysis
Source: PLoS One. 2024 Mar 7;19(3):e0298601. doi: 10.1371/journal.pone.0298601 (PMC10919601; doi:10.1371/journal.pone.0298601)
Supplement: S1 Data — (ZIP) [file pone.0298601.s001.zip › Data/地级市Z_F工作报告.zip!/╒π╜¡/╔▄╨╦╩╨/└┤╫╘╣1⁄2╓┌║┼í╛┬φ┐╦╩2╛▌═°í┐_2014.docx]

各位代表：

　　现在，我代表绍兴市人民政府，向大会作工作报告，请予审议，并请市政协委员和其他列席人员提出意见。

　　一、2013年主要工作

　　过去一年，在中共绍兴市委的领导下，市政府认真贯彻中央和省各项方针、政策，紧紧围绕市七届人大三次会议确定的目标任务，积极进取、扎实工作，保持了经济社会平稳健康发展的良好态势。全市实现生产总值3967亿元，增长8.5%；公共财政预算收入293亿元，增长10.3%；固定资产投资2002亿元，增长16.2%；社会消费品零售总额1318亿元，增长13.8%；外贸出口279亿美元，增长9.2%；城镇居民人均可支配收入40454元，农村居民人均纯收入19618元，分别增长9.6%和10.8%；居民消费价格上涨2%；城镇登记失业率2.9%；人口自然增长率0.05％。；预计研究与试验发展经费支出占生产总值比例为2.1%。其中，生产总值增幅低于预期0.5个百分点，城乡居民收入增幅分别低于预期0.4和0.2个百分点，其他主要预期目标如期完成。

　　一年来，我们牢牢把握“稳中求进、务实创新、惠民利民”工作基调，突出抓了以下六方面工作。

　　（一）力促经济平稳运行。坚持把稳增长摆在重要位置。大力推进有效投资，建立产业项目准入评估、政府投资项目监督审核等制度，加强项目建设的协调和督查，完成工业投资999.8亿元，增长13.4%；实际利用外资8.1亿美元，同比下降15.3%；引进越商回归资金159.6亿元，按同口径比上年增加35.3亿元。完善稳定出口扶持政策，组织3000多家企业参加500多场展会。制定电子商务发展规划和扶持政策，电子商务交易额99.7亿元，是上年2.1倍。改造提升农贸市场和商品交易市场，7家专业市场入选中国百强。切实加强土地、资金、人才等要素保障，消化批而未供土地2.3万亩，盘活存量土地1.5万亩，创建成为全省唯一的全国国土资源节约集约模范市；加快金融服务创新，开展金融助推转型升级五大行动，金融机构本外币贷款余额比年初增加512亿元，实现直接融资369亿元；精心举办“名士之乡”人才峰会等系列活动，引进各类人才4.8万人。

　　（二）深入推进转型升级。积极构建以十大产品为重点的现代产业体系，大力推进腾笼换鸟、机器换人、电商换市、空间换地，着力培育名企、名品、名家。全面实施战略性新兴产业“新三百推进计划”，新兴产业产值占工业总产值比重由25.6%提高到27.7%。深入实施“双百技改”工程，改造提升纺织等传统产业。扎实推进现代服务业“310工程”，实施144项重点项目，新增省级集聚示范区3家，服务业增加值占生产总值比重达到42.1%，同比提高1个百分点。积极培育新兴市场主体，完成“个转企”12314家、“小升规”616家。成功创建全国旅游标准化示范城市，启动镜湖旅游综合体、柯桥金沙·东方山水、上虞杭州湾旅游商贸综合体等项目建设，实现旅游总收入584亿元，增长15.4%。加快现代农业园区十大重点工程建设，投入资金10.3亿元。新建粮食生产功能区12.6万亩，粮食产量实现“十连增”。会稽山古香榧群被联合国列为全球重要农业文化遗产。积极推进“八倍增、两提高”科技服务专项行动，成为全省首批创新型试点城市，通过国家知识产权试点城市验收，连续九次荣获全国科技进步先进市称号。严格用能总量和强度“双控”，淘汰267家企业落后产能，单位工业增加值能耗下降7.6%。

　　（三）开启城市发展新篇。实施部分行政区划调整，撤销绍兴县、上虞市，分别设立柯桥区、上虞区，市区面积扩大到2942平方公里，人口达到216万。同步推进市直开发区体制调整，镜湖新区逐步归入越城区管理，绍兴高新技术产业开发区、袍江经济技术开发区面积大幅增加，滨海新城基础设施不断完善。杭州湾上虞经济技术开发区升级为国家级开发区。嘉绍大桥、杭甬高铁绍兴北站和上虞北站、铁路货运东站、快速公交1号线等重大工程建成投用，杭甬运河全线通航。加快推进中心镇培育，明确17个小城市培育试点镇功能定位。扎实开展美丽乡村先进县、先进乡镇、精品村和美丽农家“四级联创”，深入实施“百村示范、千村整治”，启动78个空心村改造试点，完成农房改造2.2万户。积极拓宽农民增收渠道，扶持发展农家乐、花卉等富农产业。深化区域经济协作，做好援助新疆阿瓦提等各项对口支援工作。

　　（四）整治提升城乡环境。编制城市交通治堵五年行动规划，加强道路安全设施、停车秩序和电动三轮车管理，实施交通智能化建设，严查严处交通违法行为。全面推进户外广告、矿山石料、城市扬尘等专项整治，改造旧住宅区、旧厂区和城中村1031万平方米，拆除违法建筑847万平方米。统一越城区范围征地拆迁政策，出台城中村改造高层集聚安置办法，完成老城区17个新建小区移交工作。建立工业废水排放在线监控和刷卡系统，启动污水分质提标和集中预处理，清理河道205公里，55个清水工程考核断面达标率由74.5%提高到80%。制定大气污染防治计划和应急方案，完成800多台定型机“煤改气”改造。扎实推进工业污泥、建筑泥浆、建筑渣土规范化处置。深入开展“四边三化”、“清理河道、清洁乡村”行动，完成造林更新4.6万亩。

　　（五）努力改善群众生活。全市公共财政用于民生支出227.7亿元，比上年增长13.6%，占总支出的72.9%，十方面民生实事工作基本完成。新增城镇就业11.3万人，帮助4.3万名城镇失业人员实现再就业。提高最低工资、城乡低保、医保以及企业退休人员养老金等标准，在全省率先实施城乡居民大病保险制度。开工建设保障性住房2.7万套，建成1.1万套，房地产市场保持健康发展。健全基层公共文化服务体系，开展形式多样的文化惠民活动，成功举办“两节一典礼”，成为中国书法兰亭奖永久颁奖地。推进各类教育协调发展，实施优秀教师流动制度，义务教育标准化学校创建率居全省首位，浙江工业大学之江学院、浙江农林大学暨阳学院开学招生，中职学生技能竞赛实现全省“六连冠”。公立医院改革和基层卫生综合改革成效明显，全市联网的“健康卡”正式启用，市立医院、新嵊州医院等重点工程顺利推进。广泛开展全民健身运动，成功举办国际皮划艇马拉松赛。加强小餐饮和食品药品专项整治，老城区小餐饮持证率由48%提高到91.5%。建立110社会应急联动机制。积极防御持续高温、“菲特”台风等自然灾害，有效防控H7N9禽流感疫情。妇女、儿童、老年和残疾人事业取得新进步，审计、气象、计划生育、民族宗教、外事侨务、对台事务、广播电视、新闻出版等工作取得新成绩。

　　（六）着力优化发展环境。认真贯彻中央“八项规定”精神，组织开展厉行节约、反对浪费专项行动。严格规范公务用车、考察、接待、办公用房和编外人员管理，全市“三公”经费支出下降8%，其中市级下降9.8%；公务出国（境）人数下降18%，其中市级下降36%；市级机关事业单位编外用工减少9.6%。深入开展“服务企业、服务基层”系列活动，建立机关干部“一对一”联系走访企业制度。开展企业减负“阳光行动”，减免企业各类税费13.8亿元。实施新一轮行政审批制度改革，分别向区、县（市）和市直开发区下放审批事项96项和191项，部门审批事项平均承诺办结时间缩减至4.76个工作日，提速20.5%。扎实推进“平安绍兴”建设，精心组织纪念“枫桥经验”50周年系列活动，完善信访积案化解和基层矛盾调处机制，刑事发案数和信访总量分别下降9.4%和12.7%。开展安全生产大整治，安全生产事故起数、死亡人数、直接经济损失分别下降3.7%、0.2%和2.4%。高度重视人大代表建议和政协委员提案，办理中意见采纳比例分别达到72.7%和73.6%。国防动员、国防教育、双拥优抚、人民防空和国家安全工作得到进一步加强。

　　各位代表，过去一年的成绩来之不易，这得益于历届政府打下的坚实基础，也是全市人民齐心协力、共同努力的结果。在此，我代表市人民政府，向全市人民，向给予政府工作大力支持的人大代表、政协委员、各民主党派、社会各界人士，向所有关心、支持、参与绍兴建设和发展的同志们、海内外朋友们，表示崇高的敬意和衷心的感谢！

　　各位代表，我们也十分清醒地看到，绍兴发展中还面临不少困难和问题，政府治理能力与群众期望还有一定差距。去年十方面民生实事中，提高公交分担率未达到预期目标，镜湖湿地儿童乐园未能按期开工；发展方式尚未根本转变，经济增长的质量和效益还不够高；大项目、好项目相对缺乏，发展动力有待增强；行政区划调整的后续任务十分繁重，体制理顺、规划统筹、区块融合等还有很多工作要做；基本公共服务能力还需提升，教育、医疗、住房、食品药品等方面存在不少问题，空气雾霾、河道污染、交通拥堵等群众反响比较强烈；一些地方和单位的“四风”问题仍然不同程度存在，少数机关干部担当意识不强、履职水平不高、自我要求不严。对此，我们一定高度重视，进一步坚定加快发展、科学发展、持续发展的信心和决心，不为虚名所累，不为指标所困，不为政绩所惑，坚持把群众对美好生活的向往作为奋斗目标，努力做好各项工作。

　　二、2014年的目标任务

　　今年是贯彻十八届三中全会精神的重要一年，是实施“十二五”规划承前启后的关键之年，也是绍兴行政区划调整后的开局之年。当前我们正处在转型发展的重要关口，不进则退，慢进也是退。必须牢牢把握发展大势，紧紧抓住发展机遇，主动应对各种挑战，坚持为民取向，坚持问题导向，勇于改革，勇于担当，努力开创绍兴科学发展新局面。

　　2014年工作的指导思想是：认真贯彻党的十八届三中全会精神，深入实施省委“八八战略”，全面落实市委“重构绍兴产业、重建绍兴水城”战略部署，把改革创新贯穿于各领域各环节，以提高经济发展质量和效益为中心，以提高群众满意度和幸福感为导向，着力做强城市平台，着力推进创新驱动，着力加快转型升级，着力加强社会治理，着力保障改善民生，实现经济持续健康发展和社会和谐稳定，为干好“一三五”、实现“四翻番”，加快建设现代化绍兴打下坚实基础。

　　综合考虑发展趋势和工作导向，建议2014年全市经济社会发展的主要预期目标为：生产总值增长8%以上；公共财政预算收入增长8%左右；城镇居民人均可支配收入增长8.5%；农村居民人均纯收入增长9%以上；固定资产投资增长12%以上；社会消费品零售总额增长12%；外贸出口增长7.5%；居民消费价格涨幅调控目标与省保持一致；城镇登记失业率控制在3.5%以内；研究与试验发展经费支出占生产总值比例达到2.2%；节能减排指标完成省下达任务。

　　围绕这些目标，我们将按照市委确定的“改革创新促发展、求真务实惠民生”工作基调，重点抓好四方面工作。

　　（一）以改革开放为动力促进持续健康发展。认真贯彻市委关于全面深化改革增创绍兴科学发展新优势的决定，加大重点领域、重点环节改革力度，以改革领先促进发展领先。

　　深化市场取向改革。充分发挥市场在资源配置中的决定性作用，完善公平、开放、透明的市场规则。大力发展民营经济，推进工商注册制度便利化，改先证后照为先照后证，改注册资本实缴登记制为认缴登记制。积极培育行业龙头企业，加大中小微企业扶持力度，加快“个转企、小升规、规改股、股上市”步伐。优化国有资产监管体制，健全国有企业现代企业制度。鼓励企业兼并重组，完善企业市场化退出机制。深化“亩产论英雄”理念，完善与企业综合经济效益挂钩的资源要素配置机制。积极开展城镇低效用地再开发，推行城镇土地使用税试点改革，促进土地节约集约利用。健全区域金融体系，做强地方法人银行，推动村镇银行、小额贷款公司扩面提质，力争新增全社会资金供应1000亿元，其中直接融资300亿元。积极引导金融支持实体经济发展，优化金融考评体系，提升金融服务质量。鼓励企业上市，探索建设区域性资本市场。深化省级工业转型升级综合配套改革试点，争取列入国家级转型升级改革试点城市。

　　实施创新驱动战略。健全科技投入稳定增长机制，深化“八倍增、两提高”十大专项行动，争创国家创新型试点城市。实施省级以上科技计划项目300项，新培育国家需要重点扶持的高新技术企业100家，新建省级企业研发机构30家。完善区域科技创新体系，加强政产学研合作，提升科技孵化器效率,加快中科院浙江数字内容研究院等平台建设。积极创建国家知识产权示范城市，力争专利授权量增长20%。深入实施“330海外英才计划”，启动建设国际人才村，新建2个院士专家产业化基地。

　　扩大对内对外开放。推广“政企联动、抱团参展”模式，千方百计拓展新兴市场，稳固传统市场。实施科技兴贸和品牌战略，扩大高附加值产品出口，稳定纺织、黄酒等优势产品出口。创新外贸方式，探索开展市场采购贸易，协调发展货物贸易和服务贸易，完善贸易风险应对机制。优化招商引资考核激励机制，强化产业招商和专业招商。积极引导越商回归，力争到位资金180亿元。支持企业“走出去”，开展跨国并购和技术合作。扎实推进援疆项目建设，做好山海协作、对口支援和区域经济合作工作。

　　（二）以治水为突破口加快转型升级。强化以水为核心的环境倒逼机制，重构绍兴产业、重建绍兴水城，提高经济质量、提升城市品质。

　　加快建设现代水城。科学编制绍兴水城概念性规划和重要水系专项规划，保护好“两江、十湖、一城”水体环境，打造环城河、古运河、鉴湖、曹娥江等一批精品水上景观。大力开展治污水、防洪水、排涝水、保供水、抓节水“五水共治”，深入推进“清水工程”和“双清”行动，全面推行“河长制”，积极实施污水分质提标和集中预处理，基本消灭垃圾河，有效改善臭河、黑河水质。扎实推进水城建设“双百双千”工程，实施镜湖湿地保护、迪荡湖整治、高湖滞洪区改造、曹娥江治理、白塔湖湿地保护等一批重点项目。充分发挥杭甬运河等主干道功能，推动水运复兴。制订水城旅游发展规划，加快打造全国重要的“水城旅游”目的地。

　　努力改善生态环境。在合力治水的基础上，扎实推进“清洁空气”行动，加强大气污染预警和应急响应，基本完成热电企业炉外脱硫脱硝改造，扎实开展城市扬尘、汽车尾气、餐饮油烟等专项整治，禁止农作废弃物和垃圾露天焚烧。建立健全工业污泥处置“五统一”制度，促进工业污泥、建筑泥浆、建筑渣土企业化运输、市场化运作、资源化利用。广泛开展“四边三化”，大力建设“森林绍兴”、“花卉绍兴”，积极创建国家森林城市。加快生态文明制度建设，实行最严格的源头保护、生态补偿、责任追究制度。完善生态环境功能区规划，划定生态红线。培育发展环保市场，深化节能量交易试点，扩大排污权有偿使用和交易范围。严格落实节能减排目标责任制，实行重点企业用能预算化管理。及时公布环境信息，加强社会监督。

　　积极促进产城融合。加快经济转型升级，构建与水城相适应、相融合的现代产业体系。全面推进工业强市建设，大力发展先进装备、生命健康等新兴产业，力争战略性新兴产业产值占工业总产值比重达到30%。开展“腾笼换鸟”百家企业示范创建，推进“机器换人”专项计划。实施“百企百强”、十大重点产品、企业人才“百千万”等培育工程，着力“育名企、出名品、树名家”。突出抓好绍兴纺织、诸暨袜业、嵊州领带、新昌轴承等省级产业集群转型升级示范区建设，促进传统产业高新化。坚持以产业投资带动结构调整，加强企业投资引导与服务，力争工业投资1100亿元。扩大“智慧安居”试点，促进工业化、信息化融合。弘扬城市质量精神，联动推进标准强市、质量强市、品牌强市战略。大力发展建筑工业和绿色建筑，创建“全国建筑强市”。深入推进“全市游”，分步推进“绍兴人免费游绍兴”，启动“中国兰亭”、黄酒文化园等重大旅游项目建设。进一步加大电子商务扶持力度，推动“电商换市”。培育发展文化创意、研发设计、现代物流、现代金融等新业态，着力推进健康养生特色街区、金融集聚区、通航总部基地等建设，努力促进房地产市场持续健康发展，力争服务业增加值占生产总值比重提高1个百分点。

　　加快转型升级、促进产城融合关键在企业，要坚定不移地发展实体经济，更好发挥行业协会作用，积极引导企业建立健全现代企业制度；真诚、务实帮扶企业，与企业携手共进、共渡难关；关心、关注企业经营者队伍建设，努力造就一批勇立市场潮头的企业和企业家。

　　（三）以城镇化为重点大力推进城乡一体化。切实增强中心城市辐射力、带动力，积极培育中小城市，广泛建设美丽乡村，加快形成以城带乡、城乡一体的新型城乡关系。

　　合力做强城市平台。进一步完善市与区、市与开发区管理体制，加大简政放权、充分授权力度。启动修编新一轮城市规划和土地规划，统筹市区产业和重大基础设施、公共服务设施布局。实施104国道和329国道改造、群贤路东延、绍三线北延、32省道兰亭至平水段改造等一批跨区域交通项目，加快世纪大道、展望大道、解放路、兴华路、绸缎路、兴越路等市政道路的延伸建设，促进三区的交通融合和公交一体化。实施“六湖”区域联动开发，加快绍齐公路两侧、高铁绍兴北站广场等重点区域规划建设。充分发挥国家级开发区优势，做强绍兴高新技术产业开发区、袍江经济技术开发区、柯桥经济技术开发区和杭州湾上虞经济技术开发区，提升国家级开发区和镜湖新区的城市管理、社会管理水平。统筹滨海新城各区块发展，加快江滨区开发。支持诸暨城东新城建设，力争早日形成诸暨现代城市综合中心。加快嵊州市“三江口”城市新核心区、浦口新区建设和新昌县七星新区建设，进一步促进嵊州、新昌组团式集约发展。加大店口镇、钱清镇等中心镇培育力度，形成一批各具特色、充满活力的小城市。更加科学、集约、高效地实施城中村改造，推行高层集聚安置，因地制宜开展货币化安置。深化“村改居”工作，让“村民”真正变“市民”。

　　切实加强城市管理。健全创建全国文明城市长效机制，按月开展文明指数测评。强化城市科学运营和精细管理，进一步完善城管执法体制，完成市级“智慧城管”项目建设。深化城市交通拥堵治理，完善机动车、非机动车、公交和水运“四张网”规划布局，扩大交通智能化管理覆盖面，加大超限超载等违法行为整治力度。深入开展“三改一拆”，改造旧住宅区、旧厂区和城中村400万平方米以上，拆除违法建筑300万平方米以上，启动“无违建镇村”创建。加强社区建设，规范小区物业管理，完成越城区所有失管小区移交工作。扎实推进“空间换地”，科学利用城市地下空间。

　　加大强农惠农力度。以农业“两区”为主阵地，积极推进农业现代化。加强基本农田保护和水利设施建设，保持粮食稳定增产，做强茶叶、水产、珍珠等主导产业。注重会稽山古香榧群保护利用，加大香榧等特色农产品扶持力度。大力培育专业合作社、龙头企业、家庭农场等新型农业主体，完善科技、信息等社会化服务体系，促进农业生产标准化、集约化、品牌化。建立健全农产品质量追溯体系，确保农产品安全。深化美丽乡村“四级联创”，全面启动农村生活污水综合治理，切实加强农业面源污染整治，努力提高农村垃圾集中处理覆盖面，每个乡镇至少启动1个空心村改造试点。积极壮大村级集体经济，健全土地流转机制，探索增加农民财产性收入渠道。加快推动城镇基础设施和公共服务体系向农村延伸，深入开展欠发达乡村和低收入农户奔小康工程。

　　完善城乡基础设施。健全重大项目监督审核和分类协调制度，加强项目前期工作，完善重大项目储备。做好城市轨道交通、杭绍甬高速等项目前期工作，开工建设杭绍台高速、诸暨特高压电网等重点工程，加快推进杭金衢高速绍兴段拓宽、绍诸高速诸暨延伸线、31省道绍大线北延、上虞区世纪新丘围涂、新昌钦寸水库等项目建设，确保杭长客运专线绍兴段、甬金高速嵊州互通、绍诸高速陶堰互通等项目建成投用。加强嘉绍大桥、杭甬运河、曹娥江大闸等运营管理，充分发挥工程效应。

　　（四）以改善民生为根本积极构建和谐社会。坚持民有所呼、我有所应，解决好群众最关心、最直接、最现实的利益问题，实现发展成果更多、更公平惠及全市人民。

　　繁荣发展社会事业。加强基层文化设施建设，着力构建布局合理的公共文化设施网络，广泛开展群众喜闻乐见的文化惠民活动。加大文化产业扶持力度。支持文化精品创作。从简、务实办好各类节会活动。启动学前教育新三年行动计划，开展义务教育省级特色示范学校创建，深化普通高中新课程改革，推进职业教育产教融合、校企合作，全力支持绍兴文理学院创建绍兴大学。开展“师德建设年”活动，加强教师队伍建设。深化公立医院综合改革，做好国家基层卫生综合改革重点联系点工作，建立全市统一的预约诊疗服务系统。探索卫生资源区域共享模式，促进优质医疗资源纵向延伸，提高基层医疗卫生机构服务能力。积极构建和谐医患关系，维护医护人员合法权益。鼓励社会办医、办学、办文化。完善优生促进工程，落实“单独两孩”政策。精心承办浙江省第十五届运动会，广泛开展“全民健身与省运同行”系列活动，逐步推动公共体育设施向市民开放。完善城乡就业公共服务体系，加大高校毕业生特别是困难大学生创业就业扶持力度，启动公共实训基地二期建设。提高城乡居民基础养老金和企业职工养老待遇，实施职工医疗互助保障市级统筹。完善养老事业规划，鼓励发展养老产业，积极引导社会资本兴办养老机构。加强孤残儿童、残疾人等社会救助，大力发展慈善事业。

　　改进社会治理方式。坚持发展“枫桥经验”，深化“平安绍兴”和“法治绍兴”建设。加强社会治安综合治理，完善立体化治安防控体系，打造全市“智慧天网”，依法惩治违法犯罪活动。进一步办好市长公开电话、“民情在线”和“96345公共服务热线”，建立畅通有序的诉求表达渠道。有效化解劳资纠纷，构建和谐劳动关系。深入开展“六五”普法，健全社区矫正制度，加强法律援助。优化流动人口管理和服务，让更多优秀的流动人口转变为新绍兴人。推进安全生产标准化、规范化建设，深入开展重点领域安全隐患排查，力争安全生产“三项指标”继续下降。调整食品药品监管体制，完善基层监管网络。规范互联网运用和管理，让网络空间清朗起来。完善气象灾害防御体系，提升应急管理水平，科学防控H7N9禽流感等重大疫病。加强国防动员和后备力量建设，深化国防教育，推进军民融合深度发展。继续做好双拥优抚、人民防空、国家安全、民族宗教、外事侨务、对台事务等各项工作。支持工会、共青团、妇联等开展工作。

　　切实加大民生投入。全市公共财政75%用于民生支出，重点办好十方面民生实事：（1）强化大气污染防治。严控燃煤总量，按计划完成印染企业“煤改气”工作，全面供应“国四”标准车用汽油柴油，市区空气质量优良天数不低于65%；全面推进越城区范围内19家印染企业转型升级。（2）实施治污排涝工程。市控70个水质断面中的53个达到四类及四类以上标准；实施新三江闸排涝配套河道南片拓浚工程；加快推进老小区改造、雨污分流和排涝改造工程。（3）改善城乡困难群众住房条件。新开工建设保障性安居住房16000套，竣工6000套；完成农村住房改造建设6200户，农村危旧房改造650户。（4）提升社会保障水平。启动绍兴市“市民卡”工程；实现社会保障卡全省联网就医“一卡通”；建立残疾人意外保险补贴制度。（5）加快养老服务体系建设。城市社区居家养老照料服务覆盖率达到三分之二，比去年提高1倍；新建改建农村居家养老服务照料中心430个。（6）保障食品安全。实施“绍兴市百万学生饮食放心工程”；启动放心早餐店提档升级工程。（7）缓解城市交通拥堵。实施老城区停车诱导系统；全市新增公共自行车服务网点120个、投放公共自行车6000辆；建设快速公交滨海线，市区新建296座公交候车亭，建成投用2处公交过渡性枢纽站。（8）促进创业就业。新增城镇就业10万人，高校毕业生就业率达到90%以上；引导和帮助城镇失业人员再就业3.5万人；实施创业教育和培训3.2万人次，培养技能人才5万人。（9）加强公共卫生服务。全市建成具有健康自测体检体系的健康小屋50个；实施心理健康促进工程；普及红十字现场救护知识，培训救护员13500名。（10）推进公共服务设施建设。科技中心、文化中心、奥体中心建成使用；启动新绍兴一中建设；加快市立医院建设；建成会稽山森林健身游步道50公里以上。

　　三、努力提高政府治理水平

　　认真开展政府系统党的群众路线教育实践活动，创新管理方式，着力转变作风，加快建设服务型政府和法治政府。

　　加快政府自身改革。深化行政审批制度改革，开展市级审批事项“归零清理”，推广柯桥区企业投资项目高效审批和“中介超市”经验，探索滨海新城“零审批”试点，加强审批中介机构规范管理。深化政府机构改革，稳步推行“大部门制”，优化政府职能配置，推动权力下放和人员下移。完善乡镇职能运作，提高乡村治理水平。严控机构编制和财政供养人员总量，规范编外用工管理。建立健全规范、透明的财政预算制度，加大财政预决算、部门预决算和“三公经费”公开力度，科学控制地方政府性债务。积极推广政府购买服务，提升事务性工作的质量和效率。

　　切实规范权力运行。建立法治政府实施标准和考评体系，严格规范公正文明执法。健全法律顾问制度，加强规范性文件、重大决策合法性审查。坚持重大事项向人大报告、向政协通报，自觉接受人大及其常委会依法监督，支持政协履行政治协商、民主监督和参政议政职能，广泛听取各民主党派、工商联、无党派人士和人民团体意见建议。认真办理人大代表建议和政协委员提案，改进办理方法，提升办理实效。进一步支持法院、检察院依法履职。加强对重要领域、重点环节、重大审批行为的行政监察和审计监督。健全科学民主决策和重大事项集体讨论制度，建立市级部门权力清单，扩大政府信息公开范围，让权力真正在阳光下运行。

　　全面深化作风建设。加强反腐败体制机制创新和制度保障，保持惩治腐败高压态势。进一步优化政绩考核机制，完善领导干部社会化评价制度。深入开展厉行节约、反对浪费专项行动，严格执行办公用房使用标准，规范公务接待，实施公车改革，实行公务出国（境）情况公开，全市“三公”经费预算支出削减30%。大力精简会议文电，改进文风会风；大力建设诚信政府，增强公信力、执行力；大力整治“庸懒散奢”，强化干部责任意识、担当精神；大力弘扬新风正气，积极倡导精心谋事、潜心做事、主动揽事、高效办事；大兴调查研究之风，让政府工作更合实际、更接“地气”，让人民群众更加满意。

　　各位代表！责任成就使命，奋斗铸就辉煌。让我们高举中国特色社会主义伟大旗帜，在中共绍兴市委的领导下，进一步解放思想、凝聚力量，真抓实干、开拓奋进，为加快建设经济繁荣、生活富裕、风尚文明的现代化绍兴而努力奋斗！
